# Addressing Medicine’s Dark Matter

**DOI:** 10.2196/37584

**Published:** 2022-08-17

**Authors:** Christian Rose, Mark Díaz, Tomás Díaz

**Affiliations:** 1 Department of Emergency Medicine School of Medicine Stanford University Palo Alto, CA United States; 2 Ethical AI Google New York, NY United States; 3 Department of Emergency Medicine Columbia University Medical Center New York, NY United States

**Keywords:** big data, AI, artificial intelligence, equity, data collection, health care, prediction, model, predict, representative, unrepresented

## Abstract

In the 20th century, the models used to predict the motion of heavenly bodies did not match observation. Investigating this incongruity led to the discovery of dark matter—the most abundant substance in the universe. In medicine, despite years of using a data-hungry approach, our models have been limited in their ability to predict population health outcomes—that is, our observations also do not meet our expectations. We believe this phenomenon represents medicine’s “dark matter”— the features which have a tremendous effect on clinical outcomes that we cannot directly observe yet. Advancing the information science of health care systems will thus require unique solutions and a humble approach that acknowledges its limitations. Dark matter changed the way the scientific community understood the universe; what might medicine learn from what it cannot yet see?

## Background

In this viewpoint paper, CR and his colleagues explore the limitations of current health care data and call for an acknowledgment of and action toward a more inclusive data environment.

In the early 20th century, the scientific community faced a mystifying conundrum: despite the continued growth of observational data from the most advanced measurement equipment of the time, it appeared that the mass of every visible star in the universe was not enough to keep galaxies from drifting apart. Real-world observations were not congruous with the predicted outcomes of Einstein’s Theory of General Relativity. Given that galaxies were indeed not spraying their contents across the universe but rather maintaining their place in space, something that could not be seen must have been exerting a tremendous force on the system. Now in the 21st century, the medical community faces a similar problem—a disconnect between data and outcomes. How will we uncover and address medicine’s “dark matter”? We must quantify what we are currently missing, broaden our perspective, and acknowledge our limitations.

It took a complete paradigm shift to bridge the gap between theory and observation for astrophysicists. In the 1970s, Vera Rubin and W. Kent Ford confirmed that there must be a mass at the center of galaxies that we simply cannot directly observe yet. Its existence could be inferred only by how it affected the entirety of the system. It was called “dark matter” because it did not lend itself to measurement, although it paradoxically makes up the vast majority of our universe and determines the very nature and future of our world [1].

## The Limits of Health Care Data

In the early 2000s, the Institute of Medicine noted that a similar chasm existed between the theoretical health care quality expected for our communities and the quality they actually observed. It was believed that to solve this problem, more and better health care data were needed to make more accurate predictions and provide higher quality care. Thus, since the Health Information Technology for Economic and Clinical Health Act’s enactment in 2009, the digital health landscape has grown rapidly under this assumption [2,3]. More health care data are being produced daily than ever before. Genetic test results, physiologic monitors, cell phone data, social media posts, and web searches are being incorporated into predictive algorithms.

However, if improved quality of care is the measure by which we guarantee the success of our predictions, we have little to show for our efforts [4]. Health care costs in the United States continue to increase relative to other high-income nations with minimal return on improved health care outcomes [5]. Life expectancy has remained essentially unchanged for a decade (it was actually decreasing in the United States, even before the COVID-19 pandemic) [6]. Models still fail to accurately predict health care patterns for the American population. A chasm remains between our observations and the benefits we expect from advances in health care data.

Astronomy and medicine both suffer from detection bias [7]. Researchers disproportionately value features that we can perceive and undervalue the effects we do not directly detect or understand. We have a natural preference to believe that the world we observe is the whole truth, and we exhibit little ability to think in terms of missing information. Just as the telescopes and radio receivers of astronomy were designed and used to more accurately observe the small fraction of already known entities in the universe, so too do the electronic health record, genomic sequencing machinery, and extant digital health tools make accessible data from the patient populations on which we have already focused the most resources [8,9]. The rest is medicine’s dark matter.

## That Which We Cannot See

Currently, even our largest clinical data sets contain information on only a portion of the population [8]. However, we expect data collected from this small subset to determine the course and future of our health care establishment. Gender imbalances and the underrepresentation of systematically oppressed and marginalized populations belie some of the most impactful limitations of medical data [10]. These populations rarely make it into our observations and calculations, not because they lack the need, but rather because the medical community has rarely effectively engaged them. Therefore, as we have rarely looked, we cannot see the whole truth.

The data we are missing is a reflection of our priorities. Resources for and attention to identifying and investigating even important conditions such as maternal mortality have been insufficient. Data collection varies by state, and reported statistics are incomplete [11]. Similarly, we have not prioritized diversity in clinical trials, which have historically excluded members of marginalized racial and ethnic groups [12]. The federal government acknowledged this lacuna and mandated improved representation in trials through the National Institutes of Health Revitalization Act of 1993. Nevertheless, racial and ethnic diversity among clinical trial participants remains low to this day [13]. This lack of representation limits not only the generalizability of results from clinical trials but also the potential impact of new treatments on health care quality, especially for vulnerable populations [14].

The COVID-19 pandemic has blatantly demonstrated this point. The health outcomes of the people we account for the least—the people who cannot, do not, or rarely interface with the health care enterprise—are often those at the greatest risk for poor outcomes.

## Addressing “Dark Matter”

### Quantifying What Is Missing

One solution seems to lie in making the invisible visible. If we can simply acquire the data we are missing, catalog it, and add it to our models, then we might begin to reap the benefits we expect. However, this logic is flawed [15]. If we believe that more representational data sets are a solution to this missing information, we must first address how we gain insights from communities who may already have concerns of over-surveillance or otherwise problematic visibility. As we have seen from policies such as “stop-and-frisk,” increased observations may not improve outcomes but rather worsen disparities and limit the equitable distribution of resources across communities [16]. Despite the rapid growth of artificial intelligence and its requisite data-hungry approach in health care, little attention is being given to the way data sets are collected and how this might affect the performance of the systems built upon them [17].

Even when medicine has attempted to account for these unseen populations, the proxies we use to represent complicated phenomena can be misunderstood and inappropriately related to the phenomena they approximate. This is evident in the conflation of race with racism as risk factors in medical research. Furthermore, in “Towards a Critical Race Methodology in Algorithmic Fairness,” the authors warn that “the creation of metrics and indicators which are race-like will still be interpreted as race” [18], which is to say that even as we move toward broadening our attention to those consistently left out, we must carefully consider how they are represented in data and, just as importantly, what our modeling techniques may not be able to represent about them.

Similarly, our approaches to data analysis can become barriers to better understanding. To be usable in predictive modeling, data must be quantified. Quantifying information can both allow large magnitudes of data to be efficiently processed as well as obscure the challenges underlying attempts toward the robust numerical representation of complex social processes. Classification schema may valorize certain points of view over others [19]—that is, the application of classification schema, such as census categories, can lead to trusting their validity in contexts where they may not actually be valid. This limits our attention to popular or dominant ways of categorizing data. For example, analyzing historical health outcomes of people who identify as both Black and Latino is greatly hindered or even made impossible by data collection standards that treat those categories as mutually exclusive [20].

A good place to start might be by acknowledging what we have and what is missing. Big data sets, many of which are open source, are often used to extract knowledge or train predictive models. The aim is usually to use them to improve patient outcomes, but the data they are made from are rarely assessed for generalizability or relation to the particular community of interest. Instead of blindly using them, we might explore the nature of their data and compare it to our communities. This exercise may in itself improve the knowledge of how well systems relate to each other—an internal/external audit for validity. To aid this effort, the data sets we use (and reuse) should be accompanied by robust documentation such as datasheets, which serve as a kind of nutrition label for data sets while also documenting their motivation and intended uses [21]. By cataloging the provenance of data, we can more easily assess what—and who—is missing.

### Broadening Our Perspective

With this acknowledgment of what is missing, we must then design mechanisms to solve the problem. Although more data alone will not likely solve the problem, perhaps a broader spectrum of measures can offer some short-term hope. We must begin to move beyond traditional clinical measures such as mortality, vital signs, age, or family history and include more sociocultural and even environmental data [22-24]. We now know that the risk of developing some diseases is as, if not more, reliant on an individual’s social environment rather than their genetic heritability, and yet these social determinants of health are extremely poorly captured in large data sets [25]. Perhaps there are other unknowns that we have yet to consider?

Engaging with members of the communities we seek to serve might also allow us to begin to see what we otherwise may not. An inadequately diverse representation in the medical profession is itself a barrier to patients perceiving that their own interests lie at the heart of medical research [26]. However, community members, regardless of their ties to the medical field, maintain important perspective and expertise on the questions and solutions that should be prioritized. A community-based participatory research model might help us to co-construct knowledge and build trust with communities.

Not only are these factors important independently, but they have also been proven to make the data we *do* have more accurate by their inclusion and relation to the outcomes of interest that both patients and providers alike care about, such as identifying genetic polymorphisms or predicting painful lesions from diagnostic imaging [27,28]. Similar to how binocular telescopes added depth and dimensionality to celestial images, additional perspectives to health care data might actually improve our ability to understand the realities of our patients’ experience.

### Acknowledging Our Limitations

It is possible that the solution will not lie in the data itself. Astrophysicists still cannot directly measure dark matter but that does not prevent almost every physicist from valuing it and assessing its impact on our world. Rather, it was the recognition and awareness of the biases and limitations of perception that allowed scientists to begin to account for dark matter’s immense volume and strength. When they humbled themselves to the limitations of their data, they gained insight and perspective into an even grander, more complex universe.

Here again, we might learn from our astrophysics colleagues by investigating the incongruities between model and observation. Although most studies simply impute for the missing data, perhaps we might pay more attention to why those data are missing in the first place. When the scientific community asked, “why can we not see dark matter?” the answer led to a better understanding of measurement devices and new knowledge of the effects of subatomic particles. In medicine, when we asked questions such as “why are communities of color less likely to be represented in genetic studies?” we found answers such as limited access to enrollment and mistrust in the medical community, which have solutions that are not simply related to data acquisition [29].

It was Albert Einstein himself who set the example for the scientific community, saying “We cannot solve our problems with the same thinking we used when we created them.” Medicine suffers from the expectation that it can find answers if only there were more data, more time, or more support. Perhaps a far more impactful approach would be to acknowledge the limitations of what we know and how we have come to know it and shift our focus from oversampling immense data from the patients within view and humble ourselves to reach the patients who do not come through our doors. The first step is for the medical community to look at our environment of care—our universe—through a critical lens, understanding that there is far more out there than what we have already seen.

## Key Messages

Despite decades of dedication to data collection, health care models continue to poorly predict real-world behaviors accurately. This may result from the fact that even the largest data sets only collect information from a fraction of the population, leaving large swaths of the population unrepresented and further limiting progress on health care quality. Solving this problem will require the acknowledgment of what is missing from health care data sets so that we can improve health care outcomes for all.
